# Evidence for multi-copy Mega-NUMT*s* in the human genome

**DOI:** 10.1093/nar/gkaa1271

**Published:** 2021-01-15

**Authors:** Sabine Lutz-Bonengel, Harald Niederstätter, Jana Naue, Rafal Koziel, Fengtang Yang, Timo Sänger, Gabriela Huber, Cordula Berger, René Pflugradt, Christina Strobl, Catarina Xavier, Marianne Volleth, Sandra Carina Weiß, Jodi A Irwin, Erica L Romsos, Peter M Vallone, Gudrun Ratzinger, Matthias Schmuth, Pidder Jansen-Dürr, Thomas Liehr, Peter Lichter, Thomas J Parsons, Stefan Pollak, Walther Parson

**Affiliations:** Institute of Forensic Medicine, Medical Center, University of Freiburg and Faculty of Medicine, University of Freiburg, Freiburg 79104, Germany; Institute of Legal Medicine, Medical University of Innsbruck, Innsbruck 6020, Austria; Institute of Forensic Medicine, Medical Center, University of Freiburg and Faculty of Medicine, University of Freiburg, Freiburg 79104, Germany; Institute for Biomedical Aging Research, University of Innsbruck, Innsbruck 6020, Austria; Wellcome Sanger Institute, Hinxton, Cambridge CB10 1SA, UK; Institute of Forensic Medicine, Medical Center, University of Freiburg and Faculty of Medicine, University of Freiburg, Freiburg 79104, Germany; Institute of Legal Medicine, Medical University of Innsbruck, Innsbruck 6020, Austria; Institute of Legal Medicine, Medical University of Innsbruck, Innsbruck 6020, Austria; State Investigation Department of Lower Saxony, Hannover 30169, Germany; Institute of Legal Medicine, Medical University of Innsbruck, Innsbruck 6020, Austria; Institute of Legal Medicine, Medical University of Innsbruck, Innsbruck 6020, Austria; Magdeburg University Hospital, Institute of Human Genetics, Otto von Guericke University, Magdeburg 39120, Germany; Institute of Experimental and Clinical Pharmacology and Toxicology, University of Freiburg, Freiburg 79104, Germany; DNA Support Unit, FBI Laboratory, Quantico, VA 22135, USA; U.S. National Institute of Standards and Technology, Biomolecular Measurement Division, Gaithersburg, MD 20899, USA; U.S. National Institute of Standards and Technology, Biomolecular Measurement Division, Gaithersburg, MD 20899, USA; Department of Dermatology, Venereology and Allergy, Medical University of Innsbruck, Innsbruck 6020, Austria; Department of Dermatology, Venereology and Allergy, Medical University of Innsbruck, Innsbruck 6020, Austria; Institute for Biomedical Aging Research, University of Innsbruck, Innsbruck 6020, Austria; Jena University Hospital, Institute of Human Genetics, Friedrich Schiller University, Jena 07747, Germany; German Cancer Research Center, Molecular Genetics, Heidelberg 69120, Germany; International Commission on Missing Persons, The Hague 2514 AA, Netherlands; Forensic Science Program, The Pennsylvania State University, University Park, PA 16802, USA; Institute of Forensic Medicine, Medical Center, University of Freiburg and Faculty of Medicine, University of Freiburg, Freiburg 79104, Germany; Institute of Legal Medicine, Medical University of Innsbruck, Innsbruck 6020, Austria; Forensic Science Program, The Pennsylvania State University, University Park, PA 16802, USA

## Abstract

The maternal mode of mitochondrial DNA (mtDNA) inheritance is central to human genetics. Recently, evidence for bi-parental inheritance of mtDNA was claimed for individuals of three pedigrees that suffered mitochondrial disorders. We sequenced mtDNA using both direct Sanger and Massively Parallel Sequencing in several tissues of eleven maternally related and other affiliated healthy individuals of a family pedigree and observed mixed mitotypes in eight individuals. Cells without nuclear DNA, i.e. thrombocytes and hair shafts, only showed the mitotype of haplogroup (hg) V. Skin biopsies were prepared to generate ρ° cells void of mtDNA, sequencing of which resulted in a hg U4c1 mitotype. The position of the Mega-NUMT sequence was determined by fluorescence *in situ* hybridization and two different quantitative PCR assays were used to determine the number of contributing mtDNA copies. Thus, evidence for the presence of repetitive, full mitogenome Mega-NUMT*s* matching haplogroup U4c1 in various tissues of eight maternally related individuals was provided. Multi-copy Mega-NUMTs mimic mixtures of mtDNA that cannot be experimentally avoided and thus may appear in diverse fields of mtDNA research and diagnostics. We demonstrate that hair shaft mtDNA sequencing provides a simple but reliable approach to exclude NUMTs as source of misleading results.

## INTRODUCTION

The maternal inheritance of mitochondrial DNA (mtDNA) (1) in humans stands as dogma, despite the fact that paternal leakage or a ‘doubly uniparental’ mode of transmission is well established in some animal species (2). This understanding is not only central to scientific applications in the medical, forensic and population genetic fields, but also to the investigation of human evolutionary history, including the theory of ‘Mitochondrial Eve’ (3), which holds that all modern humans derive from ancestors who lived in Africa >300 000 years ago (4,5).

Paternal leakage and recombination of human mtDNA have been proposed (6), but the underlying data and/or methods applied have been challenged (7), most of which have been discredited as artifactual (8) and extreme-depth re-sequencing of mtDNA failed to provide evidence of paternal transmission in humans (9). A highly acclaimed study observed a mixture of two mtDNA haplotypes in the muscle tissue of a male patient suffering mitochondrial myopathy (10). In the affected muscle, the authors reported a minor maternally inherited mtDNA component along with a dominant contribution matching the mtDNA sequence of the father, with the exception of a 2 bp deletion. In the patient's blood cells only the maternal mitotype was observed. Furthermore, amplicon sequencing of blood samples from the patient's mother, father, sister and a paternal uncle as well as from his sister's muscle tissue also revealed a lack of mixed mitotypes. Investigations in similar medical cases were unsuccessful in detecting or confirming any additional occurrences of paternal inheritance (11).

Recently, Luo *et al.* (12) reported phylogenetically plausible mtDNA mixtures in the blood of three unrelated patients suffering mitochondrial disorders. MtDNA analysis yielded the same mixed mitotypes also in some—but not all—closely related family members. The authors attributed the additional mitotype to ancestral fathers and claimed evidence of ‘biparental mtDNA transmission with an autosomal dominant-like inheritance mode’ (12), despite the fact that the authors did not confirm their claims by examination of the ancestral father's mtDNA. Furthermore, the authors did not determine the cellular source (mtDNA or nDNA) of the components.

In this study, we report the observation of mixed mtDNA signatures in eight of eleven maternally related individuals of a family pedigree, a constellation similar to the report by Luo *et al.* (12). In our study, however, the individuals were healthy and not aware of any pathological symptoms or disease. We investigated the cause of the mixture through carefully designed experiments that indicated the mixture was present only in cells harboring nuclear DNA. We further demonstrate that the mixtures are caused by the presence of nuclear elements of mtDNA (NUMTs) that represent full mitogenome sequences in multi-copy format (so-called Mega-NUMTs; (13)).

## MATERIALS AND METHODS

### Biological samples

**Figure 1.**
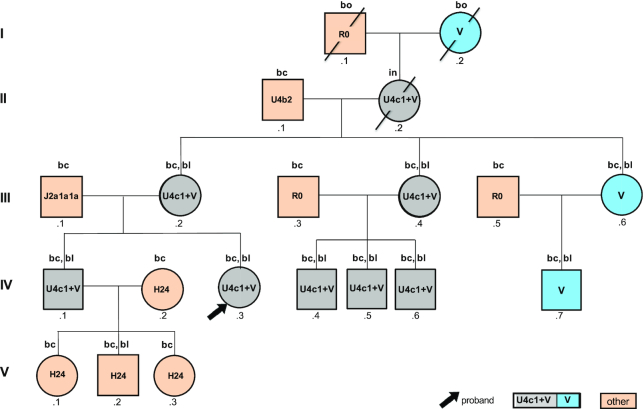
Graphical representation of the pedigree according to Bennet *et al.* (14) indicating the observed mtDNA haplogroups. Analyzed tissues are indicated above the circles and squares: ‘bc’: buccal cells, ‘bl’: blood, ‘bo’: bone, ‘in’: intestinal tissue.

All biological samples were collected under written informed consent (votes 44/10 and 319/12 of the Ethics Committee, Freiburg University, Germany). We repeatedly took samples from diverse family members in different sessions over a period of eleven years (Figure 1, Supplementary Figure S1, Supplementary Table S1). Buccal cells were taken from 17 of 20 representatives of the family. Additionally, peripheral venous blood and hairs (including roots) were collected from some of these individuals. Paraffin embedded intestinal tissue was available from the deceased maternal grandmother (II.2). Femur and cranial bones of the deceased great-grandmother (I.2) and great-grandfather (I.1) were collected during the re-establishment of the grave site. All specimens of these maternal ancestors were taken and analyzed with the approval by the family members III.2, III.4 and III.6. In addition, fibroblasts were gained from a skin biopsy taken from individual III.4.

### Measures of contamination prevention

We note here that it was critical in the early phase of the study to exclude contamination as possible source of the observed mtDNA mixtures. This is why contamination prevention is described here in detail. The confirmatory results that were obtained later (e.g. results from mtDNA sequencing of ρ^0^ cells generated from fibroblasts (15) and hair shafts, see below) serve as tangible proof that the mixture was caused by the presence of the Mega-NUMT and not by DNA contamination through an external source.

Laboratories 1 (Institute of Forensic Medicine, Freiburg, Germany) and 2 (Institute of Legal Medicine, Innsbruck, Austria) have a long-standing tradition in forensic molecular biology with particular emphasis on mtDNA analysis. They successfully participated in all proficiency testing programs of GEDNAP (16) that are required for forensic laboratories. Both laboratories are accredited according to ISO/IEC 17025 standards and use equipment and protocols that are designed and validated to prevent contamination in a forensic genetic setting. Reagent blanks and extraction negative controls were carried through the entire process to monitor potential systematic DNA contamination. Staff personnel of both laboratories were typed in all markers analyzed in this study (nuclear short tandem repeat (STR) loci, mtDNA) to serve as contamination elimination database.

Sampling of blood, buccal cells and hair took place in laboratory 1 and at the premises of the family in the course of joint meetings with the family members, during which they were also informed about the course of the analyses and their results (Supplementary Figure S1). Peripheral venous blood samples were collected in EDTA-tubes (Sarstedt, Nümbrecht, Germany) by a physician. Alternatively, cellulose tissues with dried blood were delivered by family members to laboratory 1. Blood aliquots and portions of dried blood cellulose tissues were shared with laboratory 2, where an independent analysis starting from DNA extraction was performed. Finally, we coordinated another independent round of DNA testing on a blood sample from individual IV.3 taken by a diagnostic laboratory (MVZ Clotten, Freiburg, Germany). Aliquots of this blood sample were sent in sealed envelopes to laboratories 1 and 2 for independent analyses starting from DNA extraction.

Buccal swabs of family members were obtained by the group leaders of laboratories 1 (SL-B) and 2 (WP) using ethylene oxide fumigated swabs from different companies (Sarstedt; Prionics, Zürich, Switzerland; Applimed, Châtel-St-Denis, Switzerland). For each manufacturer, devices with differing lot numbers were used for sample collection. Sarstedt and Prionics indicated that the production and the distribution of their collection devices were performed under controlled and DNA-free conditions. DNA extraction and subsequent analysis were performed at least twice on independently collected samples in both laboratories (Supplementary Figure S1).

### DNA extraction

#### Blood, buccal cells and ρ° cells

Total genomic DNA (gDNA, i.e. nuclear (n) plus mtDNA) was extracted from blood either using the QIAamp DNA Blood Mini kit (laboratory 1; Qiagen, Hilden, Germany) or using a BioRobot EZ1 Advanced XL instrument and the EZ1 DNA Investigator kit (laboratory 2; all Qiagen) following the vendor's protocols. Total genomic DNA from buccal cells was isolated using a QuickGene-810 nucleic acid isolation system and the QuickGene DNA tissue kit S (both Fujifilm, Tokyo, Japan) following the manufacturer's recommendations (laboratory 1) or with Chelex 100 (17) in laboratory 2. DNA from ρ° cells (details see below) was extracted in laboratory 2 using the procedure described for blood.

#### Hair roots and hair shafts

In laboratory 1, DNA from hair roots and shafts was extracted with the QIAamp Micro DNA kit (Qiagen) using a modified protocol (18). In brief, prior to extraction, the hair roots and shafts were sequentially washed with AL buffer (Qiagen), ethanol (80%), and HPLC-grade water for 5 min. Hair lysis was performed using 160 μl Buffer ATL (Qiagen), 20 μl 1 M dithiothreitol (DDT; Sigma-Aldrich, St. Louis, MO, USA) and 20 μl proteinase K (20 mg/ml; Roche, Mannheim, Germany) per sample with a 60 min incubation at 56°C and 800 rpm. The post lysis step was performed using 200 μl Buffer AL (Qiagen) and 1 μl tRNA (Qiagen) for 10 min at 70°C and 800 rpm. After addition of 200 μl absolute ethanol, the samples were briefly vortexed, transferred into micro spin-columns (Qiagen), washed with 500 μl AW1 and 500 μl AW2 (Qiagen), and DNA eluted in 45 μl HPLC grade H_2_O.

In laboratory 2, hair shafts were subjected to an initial mild lysis step to remove potentially non-donor related adhering biological material. For this purpose, hair shafts were completely submerged in up to 800 μl hair extraction buffer (19) containing 10 mM Tris (pH 8.0), 1 mM CaCl_2_, 100 mM NaCl, 2% sodium dodecyl sulfate (SDS; all Sigma-Aldrich) and 0.8 mg/ml proteinase K, and incubated at 56°C at 300 rpm for 2 h. Subsequently, the sample-containing tubes were briefly spun, the hair extraction buffer discarded, and the hair shafts washed thrice with fresh hair extraction buffer. Complete lysis was achieved by overnight incubation at 56°C and 300 rpm in 320 μl hair extraction buffer supplemented with 0.9 mg/ml proteinase K and 43 mM DDT. For automated DNA extraction, the EZ1 DNA Investigator kit and a BioRobot EZ1 Advanced XL instrument were used according to the large volume hair DNA extraction protocol provided by the manufacturer (Qiagen).

#### Bone

In laboratory 1, bone slices from femur (∼1.5 × 1.5 × 1.0 cm) were initially cleaned with sandpaper to remove external contaminants. Then, the samples were cut into smaller pieces (<5 mm edge length) and their surfaces were sequentially treated with HPLC-grade water, ethanol (80%), and UV-C irradiation (20 min). Bone samples were pulverized in a mill (type MM2, Retsch, Haan, Germany) using liquid nitrogen as coolant. 300 mg of the resulting fine bone powder were decalcified under constant agitation in 2 ml 0.5 M disodium EDTA (pH 8.0 at room temperature; Sigma-Aldrich) for 56 h. The EDTA was exchanged after 24 h and 48 h. After centrifugation at 600 × g for 5 min, the supernatant was discarded and the pellet was washed three times with sterile bidistilled H_2_O. Pellets were incubated in 400 μl extraction buffer (10 mM Tris–HCl, 10 mM EDTA, 100 mM NaCl, 2% SDS, 0.6% DTT, pH 8.0) supplemented with 20 μl proteinase K (20 mg/ml) under constant shaking at 56°C for 40 h. Total gDNA was extracted from this solution by following a standard phenol/chloroform/isoamyl-alcohol protocol (20). DNA was precipitated with ethanol (at a final concentration of 75%), washed with ethanol (80%) and vacuum-dried. The final pellets were solubilized in 40 μl HPLC-grade water.

In laboratory 2, bone gDNA was extracted according to the protocols detailed in (21,22). In brief, bone samples were mechanically cleaned, soaked in sodium hypochlorite solution (10%, 5 min), sequentially rinsed with molecular biology grade water and absolute ethanol and subjected to UV-C irradiation (15 min). Parts of the air-dried bone specimens were powdered with a dental drill at low speed settings. Between 100 and 400 mg of fine bone powder and an extraction blank were carefully shaken overnight at 56°C overhead in 7 ml extraction buffer (0.5 M Na_2_EDTA, pH 8.0 and 0.5% sodium *N*-lauroylsarcosinate, both Sigma-Aldrich) and 100 μl proteinase K (20 mg/ml). The tubes were centrifuged at 4000 × g for 3 min and the supernatants were subjected to DNA extraction by the phenol/chloroform/isoamyl-alcohol method (20). Centricon 30 spin filters (Merck Millipore, Billerica, MA, USA) were used to concentrate the purified DNA solutions to a volume of 100 μl by centrifugation (15–20 min at 3500 × g). The resulting retentates were further purified with the MinElute PCR Purification Kit (Qiagen) according to the manufacturer's protocol and the DNA eluted in 50–100 μl buffer EB (Qiagen).

#### Paraffin embedded intestinal tissue

In laboratory 1, paraffin embedded intestinal tissue was extracted using the RecoverAll Total Nucleic Acid Isolation kit (Thermo Fisher Scientific (TFS), Waltham, MA) according to the manufacturer's protocol.

In laboratory 2, slices from the inner portion of the paraffin blocks were cut and de-waxed in xylene (Sigma-Aldrich) at 56°C for at least 20 min. De-waxing was repeated after centrifugation in a benchtop centrifuge at maximum speed for 1 min by another xylene soak of the resulting pellet at room temperature and re-centrifugation. The pellet was washed three times with decreasing concentrations of ethanol (100%, 96%, 80%) and DNA was extracted with the BioRobot M48 workstation using the MagAttract DNA Mini Kit (both Qiagen) according to the manufacturer's instructions.

### General procedure of mtDNA and gDNA real-time PCR quantification

In laboratory 2, the mtDNA and/or gDNA content of all extracts was determined by real-time quantitative (q) PCR to ensure optimal sample input in subsequent experiments. All quantification assays were run on a 7500 Fast Real-Time PCR System (TFS) using the instrument's standard temperature ramp-speed setting. Genomic DNA contents were determined by qPCR amplifying a ∼71 bp long part of the multi-copy Alu Yb8 elements (23) as detailed in (24) with modifications (25). For mtDNA quantification, a 143 bp mitochondrial sequence (26) was co-amplified with a non-competitive PCR positive control system, following the protocol published by (27). Both amplification primers and the hybridization probe used for mtDNA amplification and detection had perfectly matching binding sites on both mitotypes under study. MtDNA quantification results were expressed as ‘mitochondrial genome equivalents’ (mtGE; one mtGE equals one double-stranded template molecule).

In experiments to estimate the Mega-NUMT copy number in mtDNA-free cultured fibroblasts (see section ‘Experiments on ρ° *cells*’ and Supplementary Figure S1), nDNA and mtDNA quantification was also performed simultaneously in laboratory 2 with a quadruplex qPCR assay (28), which was not available in the first round of general quantification experiments. For details see section ‘NUMT insert copy number estimation in DNA obtained from ρ° cells’.

### Confirmation of kinship and contamination testing by autosomal short tandem repeat genotyping

In laboratory 1, all samples under study were subjected to STR genotyping using the PowerPlex16 kit (Promega, Madison, WI, USA) according to the manufacturer's recommendations. Likewise, in laboratory 2, the PowerPlex ESI 17 System (Promega), Identifiler Plus and Minifiler kits (both TFS) were applied according to the manufacturers’ recommendations. Fluorescent electrophoretic sizing of the PCR products was performed on ABI Prism Genetic Analyzer 3130xl (laboratory 1) or 3100 (laboratory 2) instruments using default settings with Data Collection Software v3.0 and analyzed using GeneMapper ID v3.2 (laboratory 1) or GeneMapper v3.7 (laboratory 2) (all TFS).

### Experiments on buccal cells, blood, bone, hair and intestinal tissue

#### MtDNA amplification and direct sequencing of PCR products

In both laboratories the entire mtDNA control region (CR) was sequenced after 32 and 35 cycle PCR amplification, respectively, by using primers F15900/R599 (Supplementary Table S2) and following the procedures described in (29). However, in deviation from the published protocol, 1× Advantage 2 polymerase mix (Takara Bio Europe, Saint-Germain-en-Laye, France) was used instead of AmpliTaq Gold DNA polymerase (TFS). Direct fluorogenic Sanger-type cycle-sequencing of the PCR products was performed as described in (29). In laboratory 1, these experimental conditions were also used to amplify the entire CR with primers F15851/R639 and the resulting PCR products were sequenced with primers F15851, F16268, F29, R159 and R639 (Supplementary Table S2). Capillary electrophoretic separation of cycle sequencing products was carried out on ABI Prism Genetic Analyzer 3130xl (laboratory 1) or 3100 (laboratory 2) instruments, each equipped with a 36 cm capillary array and POP6 as sieving matrix (all TFS). For raw-data acquisition, the ABI PRISM Genetic Analyzer Data Collection software was used (v3.0 for 3130xl, v2.0. for 3100; both TFS). In both laboratories, mtDNA sequence data were phylogenetically aligned (31) and compared to the ‘revised Cambridge Reference Sequence’ (rCRS) (30,32) with Sequencher software (Version 4.9, GeneCodes, Ann Arbor, MI) following the nomenclature guidelines for mtDNA typing (33–37).

In laboratory 1, this experimental workflow was applied to gDNA extracts from buccal cells, blood, bone samples, intestinal tissue and hair gDNA; Amplification with alternative PCR primers (F15851/R639, Supplementary Table S2) was performed for additional CR sequencing on buccal cell and blood samples.

In laboratory 2, amplification of the entire CR with primers F15900 and R599 (Supplementary Table S2) (29) was restricted to high-quality gDNA extracts from blood and buccal cells (38). For mtDNA from bone and paraffin embedded intestinal tissue two PCR ‘tiling’ approaches (39) were used for sequencing analyses, as they reduced the quality-requirements for the mtDNA templates by amplifying the entire CR in the form of multiple overlapping mid-sized or short-length PCR products. The experimental procedures for multiplexed PCR amplification and direct Sanger-type sequencing followed the protocols published in (38,39).

Moreover, in laboratory 2 full mitogenome sequencing was performed using two different strategies. (i) Long-range PCR amplification of the overlapping regions 2480–10858 and 10653–16569|1–2688 (40) was performed according to (41). This particular PCR strategy was applied to gDNA extracted from venous blood of individual IV.3 and from mtDNA-free cultured fibroblasts (individual III.4, see section ‘Experiments on ρ° cells’). (ii) Next Generation Massively Parallel Sequencing (MPS) was performed using the Precision ID mtDNA Whole Genome Panel (TFS) on an Ion S5 instrument following the manufacturer's recommended protocol that is described in detail in (42).

#### Cloning analyses

In laboratory 1, purified (QIAquick PCR purification kit, Qiagen) PCR products obtained from blood and buccal DNA (individuals III.2, III.4, III.6, IV.1 and IV.3–7), from intestinal tissue (individual II.2), and from bone (individuals I.1 and I.2) were cloned in chemically competent One Shot *Escherichia coli* bacteria using pCR2.1 or pCR4-TOPO-TA vectors (all TFS). Alternatively, JM109 *E. coli* bacteria and the pGEM-T vector (Promega) were used for this purpose according to the manufacturer's recommendations. The inserts covered at least positions 70–300. Bacteria were grown overnight at 37°C on agar plates containing ampicillin (50 μg/ml; Roche) and X-Gal (60 μg/ml; TFS) for selection. At least 96 colonies were picked, directly amplified and sequenced or incubated overnight in 1.25 ml LB medium (Carl Roth, Karlsruhe, Germany) containing ampicillin (50 μg/ml) at 37°C. Plasmid minipreps were performed with the Perfectprep Plasmid 96Vac Kit or Fast Plasmid Mini Kit (both 5-Prime GmbH, Hamburg, Germany). Sequencing of cloned CR segments (29) spanning at least positions 70–300 was performed using 2 μl of the purified plasmid DNA in a total volume of 10 μl, and with electrophoretic separation and detection on an ABI PRISM 3130xl Genetic Analyzer using default conditions.

### Analyses of peripheral blood mononuclear cells and single thrombocytes

#### Preparation and sorting

In laboratory 1, peripheral blood mononuclear cells (PBMC) from individuals III.2, III.4 and III.6, IV.1 and IV.3 and single thrombocytes from individual IV.3 derived from peripheral blood samples were isolated using Leucosep centrifuge tubes (Greiner Bio-One, Frickenhausen, Germany; Figure 1, Supplementary Table S1). Single cells were deposited in individual wells of 96-well plates (ABgene, Hamburg, Germany) using a MoFlo cell sorter (Beckman Coulter, Brea, CA). PBMCs were sorted using forward (low angle, FSC) versus side scatter (90° scatter, SSC) signals to distinguish between live and dead cells, debris and other cell types. The area of forward scatter versus pulse width was used to distinguish between single cells and doublets. The thrombocytes were first stained with anti-human CD42a FITC antibodies (Becton Dickinson, Franklin Lakes, NJ, USA) and then sorted using forward (low angle, FSC) logarithmic versus side scatter (90° scatter, SSC) logarithmic signals. We then used FSC log versus pulse width to separate single thrombocytes versus doublets, followed by a narrow gate on CD42a^+^ FITC to select the labeled thrombocytes.

#### PCR amplification and direct sequencing of mtDNA targets

In laboratory 1, amplification of mtDNA from single PBMCs and single thrombocytes was carried out in 96-well plates as received from the respective deposition techniques without prior DNA extraction.

36-cycle PCR amplification of PBMC and single thrombocyte DNA with primers F15/R381 or F15851/R639 (Supplementary Table S2), purification of the amplicons, cycle-sequencing with primer F29 (Supplementary Table S2) and post-sequencing manipulations were performed as described in ‘MtDNA amplification and direct sequencing of PCR products’ (laboratory 1).

### Experiments on ρ° cells

#### Preparation of the primary fibroblasts from skin biopsies

Skin biopsies (∼5 × 5 × 5 mm) were taken from the upper medial arm of donor III.4 and an unrelated person who served as a control. These experiments took place at the Institute for Biomedical Ageing Research, Innsbruck, where the samples were immediately rinsed with phosphate buffered saline (PBS, pH 7.4) and incubated for 30 min in Dulbecco's modified Eagle's medium (DMEM, TFS) supplemented with penicillin (500 IU/ml), streptomycin (500 μg/ml) and Fungizone (12 μg/ml, all three Sigma-Aldrich). The dermis was separated from the epidermis after an overnight incubation at 37°C in DMEM supplemented with dispase (2.5 mg/ml, Roche), penicillin (100 IU/ml), and streptomycin (100 μg/ml). The hence separated dermis was then incubated at 37°C for 8 h in DMEM containing collagenase A (1.5 mg/ml, Roche), dispase (2.5 mg/ml), penicillin (100 IU/ml), and streptomycin (100 μg/ml). To arrive at a single cell suspension, the obtained solution was filtered twice with a 100-μm cell strainer (Sigma-Aldrich). The cells were counted and resuspended for expansion in DMEM containing 10% fetal bovine serum (FBS), 2 mM l-glutamine (both TFS), 100 IU/ml penicillin and 100 μg/ml streptomycin. Skin fibroblasts were maintained in DMEM as described before (43).

#### Generation of ρ^0^ skin fibroblasts

The generation of mtDNA-free skin fibroblasts (ρ cells (15) followed a protocol described previously (44). Primary fibroblasts from the skin biopsies were maintained in high glucose DMEM supplemented with 10% FBS, 2 mM l-glutamine, 100 μg/ml sodium pyruvate (TFS) and 50 μg/ml uridine (Sigma-Aldrich) and passaged by trypsinization twice a week to avoid confluency. Twenty-four hours after plating the cells to a 10-cm Petri dish (1 × 10^5^ cells/dish), 0, 50, 75 or 100 ng/ml ethidium bromide (EtBr; Sigma-Aldrich) were added to the culture medium. Around 7–14 days after the addition of EtBr, when dead cells began to peel off from the dish (dependent on the EtBr concentration), the plates were washed once with PBS to remove dead cells and fresh medium was added with EtBr at the appropriate concentration. To monitor pH, the medium was changed each time, when the medium began to appear yellow. The cultures were subsequently expanded in DMEM, supplemented with EtBr and after four months of cell culture, when PCR data confirmed lack of mtDNA, the fibroblasts were trypsinized, washed with PBS, resuspended in 1 ml PBS and stored at –80°C until further analysis.

#### mtDNA sequencing analysis on ρ° cell gDNA

MtDNA sequencing analysis of ρ° cell gDNA (laboratory 1 and 2; Supplementary Table S1) followed the routines described in section ‘MtDNA amplification and direct sequencing of PCR products’.

#### NUMT insert copy number estimation in DNA obtained from ρ° cells

The Mega-NUMT:nDNA copy number ratio was estimated with droplet digital PCR (ddPCR; performed at the Biomolecular Measurement Division of NIST, Gaithersburg, MA) on four mtDNA and one autosomal target sequences as detailed in (45). The amplified rCRS ranges were chrM:3485–3553 (‘ND1’, 69 bp, (46)), 13288–13392 (‘KAV’, 105 bp, (47)), 8446–8524 (‘BATZ’, 79 bp, (24)) and 8294–8436 plus chr1:633463–633605 (‘AND’, 143 bp, (26)). The autosomal target sequence (‘ND14’) encompassed GRCh38 positions chr14:94841905–94842013 (109 bp, (45). For data analysis, all target molecules were assumed to be double-stranded. The number of mtGEs per ρ° cell was determined by multiplying the mean apparent Mega-NUMT:ND14 ratio by a factor of 2, to account for the diploid nature of the cultured fibroblasts. To verify the ddPCR results in question, a multiplexed real-time qPCR assay described in (28) was additionally used in laboratory 2, which co-amplified a synthetic PCR positive control system, two mtDNA sequences (ND1, AND) and an autosomal multi-copy target (‘RNU2’) (48,49). Total gDNA extracted from nucleated blood cells (buffy coat) of a single healthy human female donor (SRM2372a, component B, NIST, Gaithersburg, MD, USA) served as metrologically valid, certified mtDNA and gDNA real-time PCR quantification standard with known mtDNA:nDNA ratio (45).

### Chromosome preparation and fluorescence in situ hybridization

These experiments were conducted at the Institute of Human Genetics, Jena, Germany. Metaphase spreads were obtained from peripheral heparinized blood of individual IV.3 by standard procedures (50). Briefly, T-lymphocytes were cultured in Roswell Park Memorial Institute medium (Sigma-Aldrich) for three days at 37°C and 5% CO_2_ in a humidified incubator. Cells were stimulated to leave G0-phase by phytohemagglutinin (Roche). To cause metaphase arrest, colcemid (10 μg/ml, TFS) was added 2 h before harvesting. Harvested cells were subjected to hypotonic shock (in 75 mM KCl) to separate chromosomes from each other and then suspended in fixative (methanol/acetic acid 3:1) and stored at –20°C. The cell suspension was spread on microscope slides and air dried (51).

Fluorescence *in situ* hybridization (FISH) experiments were performed as described previously (50). Briefly, a ∼8.5 kb long-range mtDNA PCR product (see section ‘MtDNA amplification and direct sequencing of PCR products’) was amplified from individual IV.3 and used as probe. In addition, we also used a centromeric probe specific for chromosomes 14 and 22 (D14/22Z1; Cytocell, Cambridge, UK). The latter was directly labelled with Anti-Digoxigenin-Rhodamin (Roche; red signal). The mtDNA probe was indirectly labelled with Biotin-16-dUTP (Roche) by using two consecutive rounds of degenerate oligonucleotide-primed polymerase chain reaction (DOP-PCR – for details see (52). 0.25 μg of mtDNA probe, together with 2.5 μg of COT1 DNA were diluted in 7.5 μl of hybridization mix (Cytocell), denatured at 95°C and pre-hybridized for 20 min at 37°C. In parallel, 0.5 μl of D14/22Z1 probe diluted in 2.5 μl of hybridization mix (Cytocell) was denatured at 95°C and stored at 4°C. Also, the slides with spread chromosomes were denatured at 75°C using a standard denaturation buffer (50). Then, both probe solutions were mixed and the overall 10 μl were added to the denatured slides and hybridized overnight at 37°C in a humid chamber. After post-hybridization washes in 0.1× SSC/Tween (3 × 5 min) the mtDNA probe was detected by Streptavidin-FITC (Roche) leading to green FISH-signals. Images of 25 metaphases were captured using an Axioplan 2 microscope (Zeiss, Jena, Germany) using the ISIS software (Metasystems, Altlussheim, Germany).

## RESULTS

### Confirmation of kinship and exclusion of contamination

Autosomal STR typing of all family members confirmed the expected kinship (Figure 1, Supplementary Figure S2) and did not reveal any indication of a mixture at the nDNA level. This excludes external contamination of the DNA extracts that were also used for mtDNA sequencing. Both laboratories used different STR kits, however, the shared STR markers yielded identical results. None of the STR genotypes matched any of the entries of both laboratories’ contamination elimination databases. Also, the haplogroup (hg) U4c1 mitotype, which corresponds to the Mega-NUMT sequence, was not found in these databases. Furthermore, the results of repeated analyses within and between laboratories were fully consistent across individuals and tissues (Supplementary Figure S1 and Supplementary Table S3). Finally, the samples collected and shipped by an independent commercial laboratory (MVZ Clotten) also confirmed the STR genotypes mentioned above, as well as the mtDNA sequencing data described below. Negative controls and extraction blanks were carried through the laboratory process and did not result in STR genotypes or mitotypes.

### Sequencing of pedigree members

**Figure 2.**
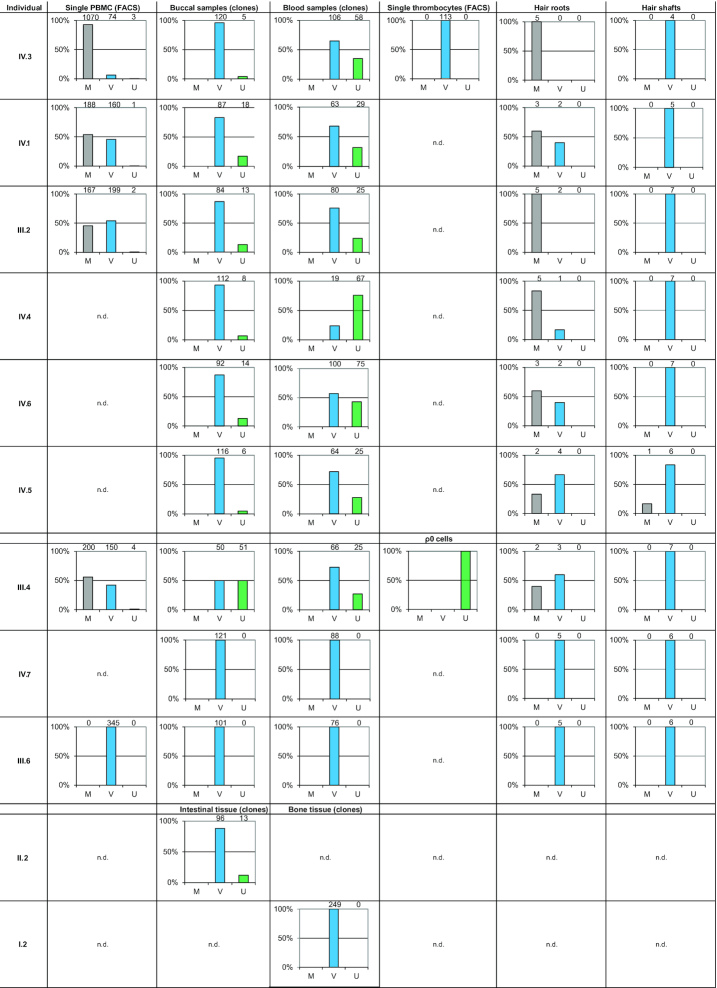
Bar charts representing the observed mtDNA CR sequencing results from single PBMCs (Peripheral Blood Mononuclear Cells) and thrombocytes (both separated by single cell FACS), single hair roots, single hair shafts and ρ° cells, as well as single clones generated from buccal cells, blood, bone and intestinal tissue samples. The height of the bar relates to the number of observations, whereas the numbers above the plots indicate the absolute count of analyzed cells or clones. ‘M’: mixed sequence of both the hg V mitotype (V) and the hg U4c1 mitotype (U), ‘n.d.’: not detected.

Direct Sanger sequencing of PCR products of gDNA extracted from venous blood of individual IV.3 resulted in the mixed mitotype 16179Y 16298Y 16356Y 16512Y 16519Y 72Y 73R 189R 195Y 200R 263G 315.1C 499R (Table 1, Figure 1). Assuming two contributing sources, the deconvolution of the mixture following phylogenetically established mutation patterns resulted in hg HV0 (16298C 72C 200G 263G 315.1C) and hg U4c1 (16179T 16356C 16512C 16519C 73G 189G 195C 263G 315.1C 499A) CR mitotypes (53). Sanger sequencing of cloned PCR products yielded dominant hg HV0 (96% and 65%) and minor hg U4c1 mitotypes (4% and 35%) for DNA extracted from buccal cells and blood samples from individual IV.3, respectively (Figure 2). Whole mitogenome sequencing was performed on DNA extracted from blood of individual IV.3 using two different approaches, (i) long-range PCR and Sanger sequencing with internal primers (41) and (ii) Next Generation Massively Parallel Mitogenome Sequencing (MPS) using a primer tiling assay (42). Both methods yielded identical mixture results that also matched the CR mitotypes obtained by Sanger sequencing mentioned above (Table 1). The coding region sequencing results were also identical and yielded a mixture (assuming two contributors) of phylogenetically plausible hg V (nested in HV0) and U4c1 full mitogenomes, thus confirming and extending the earlier CR results (Table 1, Supplementary Table S4; these two mitotypes are further referred to as U and V throughout the manuscript).

**Table 1.** MtDNA control region (CR) sequences of eleven maternally related family members and the mtDNA coding region (codR) sequence of individual IV.3. MtDNA CR sequences span rCRS (30) nucleotide positions (ntps) 16024–576 and were generated from blood, buccal, bone and intestinal tissue samples. The mtDNA codR sequence (ntps 577–16023) was generated from a blood sample of individual IV.3. by direct sequencing of PCR products. In buccal cells and the intestinal tissue the hg V mitotype (nested in hg HV0) was the quantitatively dominant type, whereas the hg U4c1 (U) mitotype was present as the minor component at the limit of detection for Sanger sequencing (<20%; Figure 1 and Supplementary Figure S3). In blood, the U mitotype generally exceeded the lower detection limits. Individuals IV.7, III.6 and I.2 showed only the V mitotype in blood, buccal cells and bone. Direct CR and full mitogenome long-range PCR Sanger sequencing of the DNA extracted from ρ° cells of individual III.4 exclusively yielded the U mitotype. Signature variants for hg estimation are color-coded: hg V (HV0) – blue; hg U (U4c1) – green

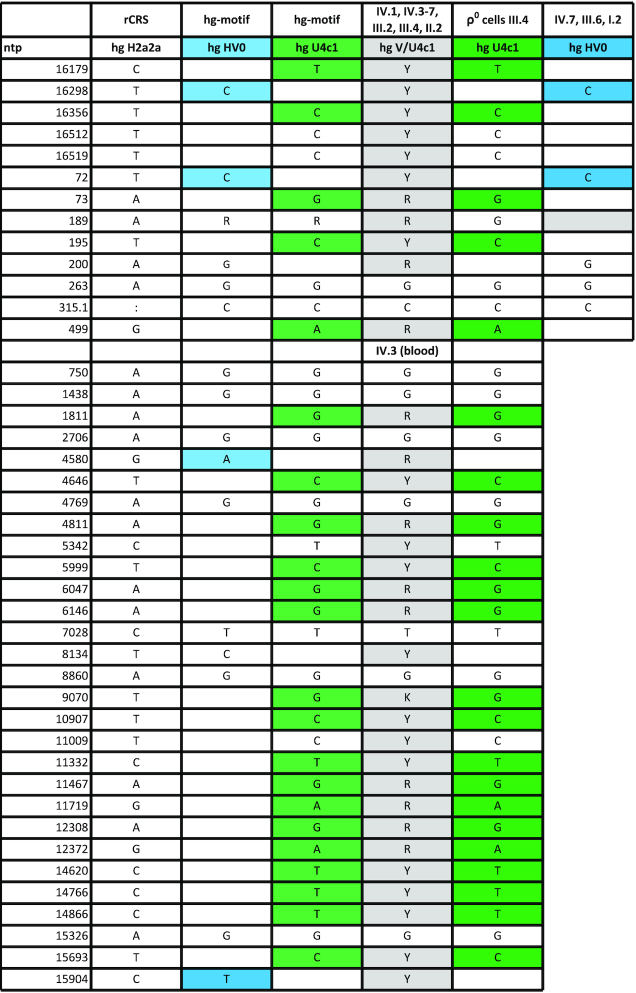

MtDNA analysis was extended to a total of 19 additional family members, of which 10 were maternally related to individual IV.3 (Figure 1). Sanger sequencing of CR amplicons derived from gDNA showed this mixed mitotype in blood and—less pronounced—in buccal samples provided by six maternally related family members of individual IV.3, i.e. her brother (IV.1), her mother (III.2), her aunt (III.4) and her three cousins (IV.4–6; Table 1, Figure 1, Supplementary Figure S3). Furthermore, the mixed mitotype was also observed in an intestinal sample from her deceased grandmother (II.2; Table 1, Figure 2). In contrast, her aunt (III.6), her cousin (IV.7) and her deceased great-grandmother (I.2) harbored only the V mitotype in blood, buccal cells and bone tissue, respectively (Figure 1, Table 1). The remaining pedigree members outside this maternal lineage, i.e. individuals I.1, II.1, III.1, III.3, III.5, IV.2, V.1-.3 yielded different single-source mitotypes that were all different from the mitotypes presented above. On the basis of these data, neither individual I.1 nor I.2 were identified as the source of the U mitotype in the family tree examined here (Figure 1, Supplementary Table S3) (see discussion).

### Confirmatory sequencing of cloning and FACS products of selected tissues

Blood and buccal samples as well as intestinal tissue and bone samples from maternally related individuals I.2, II.2, III.2, III.4, III.6, IV.1 and IV.3–7 were further subjected to cloning analysis. The sequencing results of the clones confirmed the above described mixture in all samples. Also, the lack of the U mitotype was confirmed by cloning experiments in individuals I.2 (bone) and III.6, IV.7 (blood and buccal cells) that exclusively resulted in the V mitotype. Sanger sequencing of 249 clones developed from the bone tissue of I.2 did not reveal any U contribution (Figure 2). The cloning analyses of the bone sample of I.1 also yielded no U mitotype (data not shown).

PBMCs were isolated from peripheral venous blood of individual IV.3, her brother (IV.1), her mother (III.2) and her aunts (III.4, III.6). CR Sanger sequencing of several hundred single cells per individual confirmed the mixture observed with direct sequencing of whole blood in these individuals and also confirmed the findings that aunt III.6 harbored only the V mitotype (Figures 1 and 2, Supplementary Figure S3).

### Sequencing of tissues with reduced nuclear content

Single thrombocytes, void of nuclei, were isolated from peripheral venous blood of individual IV.3 and extracted for direct Sanger sequencing. In contrast to the observed U/V mixture in whole blood of IV.3, only the V mitotype was detected in a total of 113 isolated thrombocytes (Figure 2) suggesting that the U mitotype was not originating from the mitochondria. Hair samples were taken from all nine living individuals of the matriline and the root portions were excised for separate analyses. Direct sequencing of hair shaft extracts (that typically lack high molecular weight nDNA) predominantly yielded the V mitotype (in 55 of 56 analyzed samples; 4–7 samples per individual; Figure 2). Only one hair shaft showed the U/V mixture in individual IV.5, of whom a total of seven hair shafts were sequenced. These results confirm the findings observed in single thrombocytes with the single exception of the mixture in IV.5, which we attribute to some nucleic remnants in that sample. In contrast, the root portions of the corresponding hair samples all yielded the U/V mixture except for individuals IV.7 and III.6 that—in agreement with direct CR sequencing of blood and buccal cells—resulted only in the V mitotype (Figure 2). Hair roots also yielded pure V mitotypes in individuals III.4 (3 out of 5), IV.1 (2 out of 5), IV.4 (1 out of 6), IV.5 (4 out of 6) and IV.6 (2 out of 5), which may be caused by an excessive mtDNA to nDNA ratio in these samples. Furthermore, short amplicon MPS on independently taken hair shaft samples yielded only the V mitotype in all nine investigated individuals (Supplementary Table S4). In conclusion, the results from the hair analyses further corroborate the assumption that the U contribution did not derive from the mitochondria.

### Sequencing of ρ^0^ cells

The hypothesis that the U mitotype did not originate from the mitochondria was subjected to a more substantive test by analyzing ρ° cells (depleted of mtDNA) that were prepared from a skin biopsy of individual III.4, who showed the U/V mixture in blood and buccal cells. CR Sanger sequencing of the ρ° cell DNA exclusively yielded the U mitotype, providing a strong proof that the U mitotype indeed derived from nDNA (Table 1, Figure 2). This finding was confirmed by Sanger sequencing results obtained for the two overlapping long-range PCR products, which cover the sequence of the entire human mitochondrial genome. According to these results the source of the U mitotype corresponded to nuclear mitochondrial-like sequences (NUMTs), while the V mitotype was exclusively observed in samples containing mitochondria and thus represents the authentic mtDNA in this matriline.

### Nuclear location and copy-number of the U mitotype

**Figure 3.**
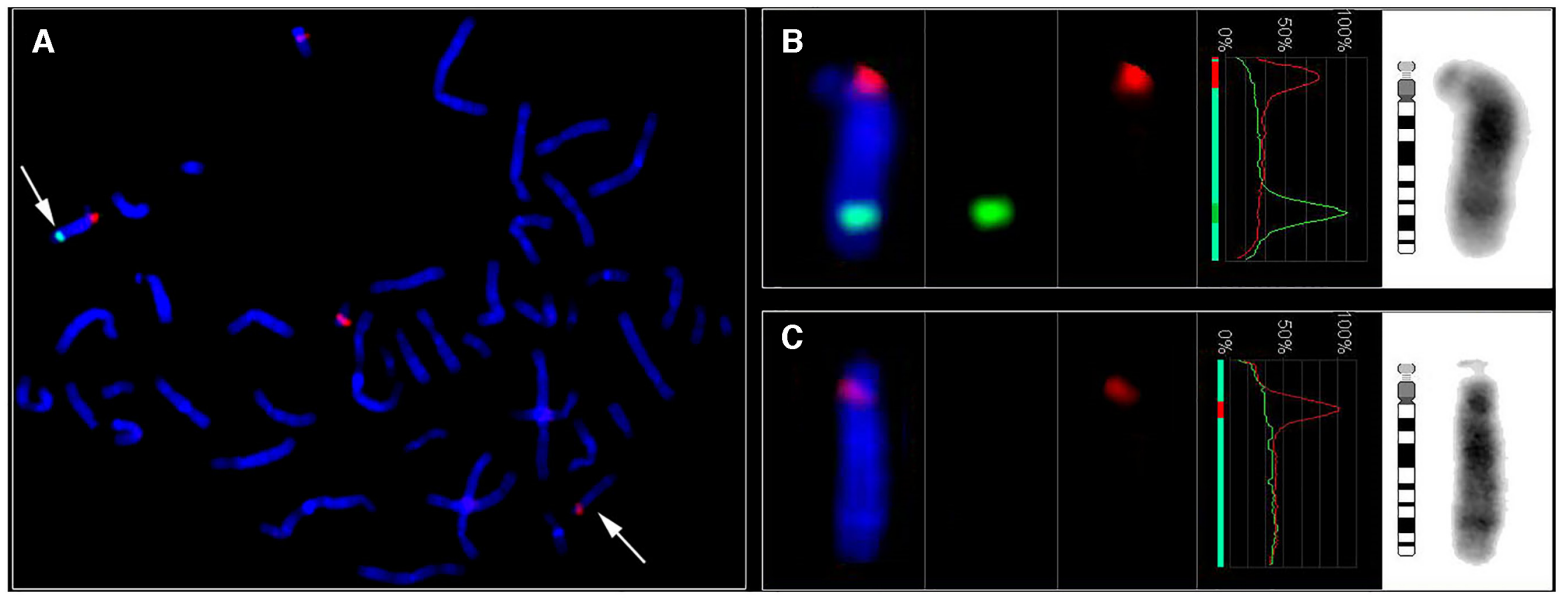
Characterization of the mtDNA insertion (Mega-NUMT) on chromosome 14. (**A**) Metaphase FISH generated with the Metasystems (ISIS) software show the Mega-NUMT on chromosome 14 (green, FITC signal, arrows). Centromeric FISH probe for chromosomes 14/22 was applied as a control (red, Rhodamin signal). Accordingly, this FISH probe shows specific signals on both chromosomes 14 and co-hybridization signal in the centromeric part of both chromosomes 22. (B and C) Detailed results per chromosome using an overlay of the green and red color channels, the fluorescence intensity profile along the chromosome, and the inverted DAPI image. (**B**) Derivative chromosome 14 containing the Mega-NUMT insertion at 14q31. (**C**) Homologue chromosome 14 without the NUMT insertion.

**Figure 4.**
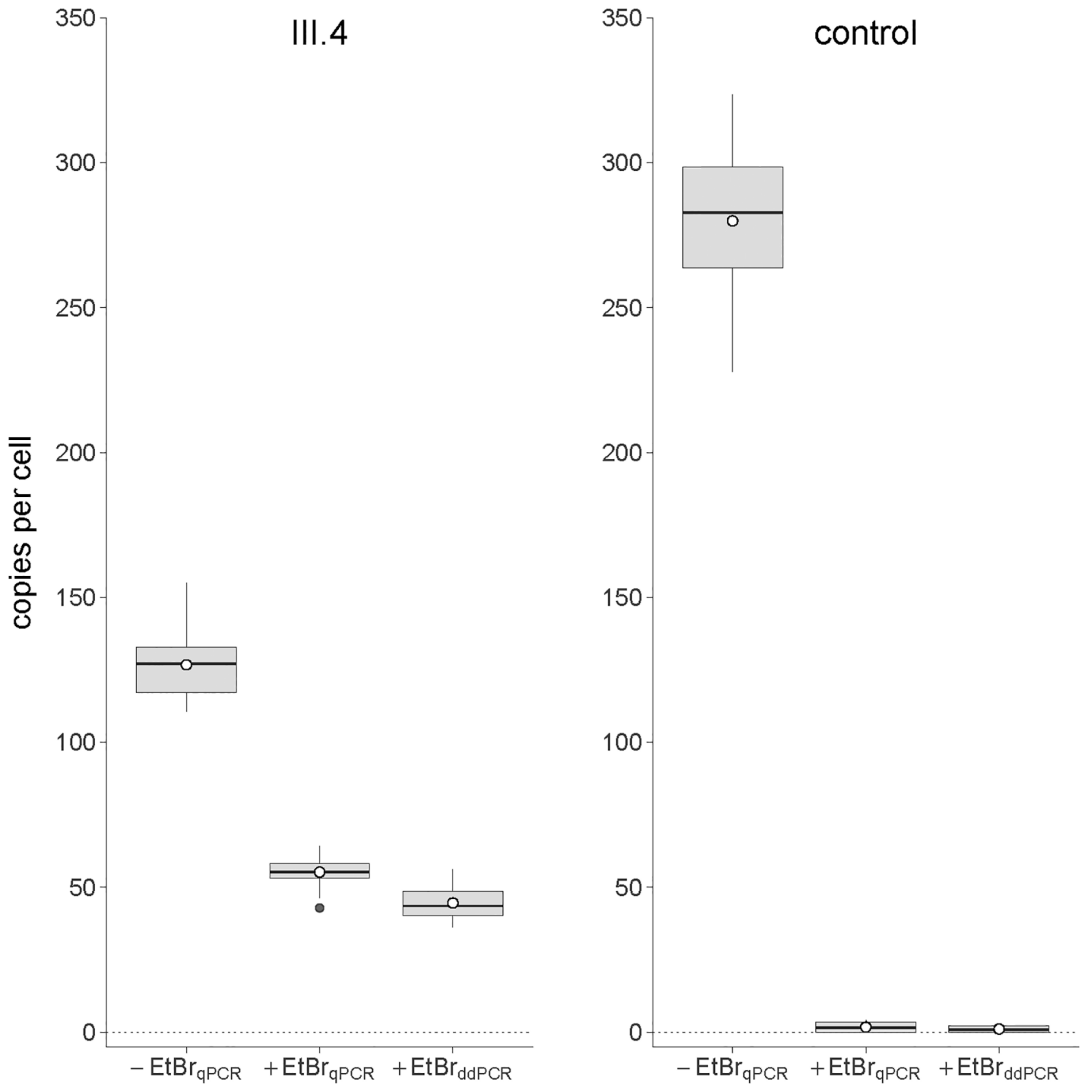
Estimated number of mtDNA building blocks of the chromosome 14 Mega-NUMT. The ostensible mtDNA copy number per NUMT was estimated by droplet digital PCR (45.00 ± 5.27; *n* = 20) and real-time PCR quantification (56.16 ± 4.35; *n* = 76) in ρ° cell DNA extracts. The results obtained for fibroblasts of an unrelated individual (control) demonstrated efficacy of ρ° cell culture. Information contained in here summarizes data on four (ddPCR) or two (qPCR) mtDNA and one nDNA quantification target sequences and four different concentrations of ethidium bromide (0, 50, 75 and 100 ng/ml) applied to ρ° cell cultures. The lower/upper hinges of the boxes indicate the first/third quartiles (Q1/Q3), horizontal lines within boxes denote the median and the whiskers extend from Q1/Q3 to the minimum/maximum values in the dataset or, in the presence of outliers, from Q1/Q3 to the upper/lower fence of the distribution (i.e. Q1 – 1.5 × IQR, Q3 + 1.5 × IQR; IQR = Q3 – Q1). Potential outliers are shown as individual black dots below/above whiskers and the arithmetic mean is indicated by a white dot with black border. -EtBr: standard cell culture conditions without addition of ethidium bromide; +EtBr: ρ° cell culture with ethidium bromide (combined data for 50, 75 and 100 ng/ml culture medium); qPCR: real-time quantitative PCR results; ddPCR: droplet digital PCR results.

FISH and two different mtDNA quantification methods were used to determine the chromosomal location and copy-number of the U mitotype. FISH located the NUMT at chromosome 14q31 (Figure 3) using a blood sample provided by individual IV.3. Droplet digital PCR was used to estimate the number of mtDNA inserts forming the Mega-NUMT sequence on chromosome 14. To simplify this task, gDNA was extracted from mtDNA-free fibroblasts (ρ° cells) (15) from individual III.4, which were exposed to different concentrations of EtBr during cell culture. Even the fibroblasts subjected to the lowest concentration of EtBr (50 ng/ml) showed a substantial loss of mtDNA relative to EtBr-free controls, The mtDNA content did not further decrease with higher EtBr concentrations (up to 100 ng/ml; data not shown). Based on the combined ddPCR data, we arrived at an estimate of 45.00 ± 5.27 (*n* = 20) U mitotype copies per Mega-NUMT (Figure 4). To cross-check these ddPCR results, we subjected the same DNA extracts to single-tube gDNA/mtDNA quantification by means of a quadruplex real-time qPCR assay (28). This approach yielded 56.16 ± 4.35 (*n* = 76) mitochondrial genome equivalents per Mega-NUMT (Figure 4).

## DISCUSSION

‘Mitochondrial–DNA-like sequences’ were described in humans and other species already in 1983 ((54) and references therein), shortly after the first human mitogenome sequence was published (32). The term ‘nuclear mtDNA segment’, short NUMT, was coined by Lopez *et al.* in 1994, who observed the transposition of a 7.9 kb mtDNA fragment into the nDNA of the domestic cat (55). Ten years later a comprehensive study revealed the presence of a plethora of NUMTs in the human genome, including almost full mitogenome sequences (56). Since then and particularly with the emergence of MPS technologies NUMTs have been regularly described in studies investigating mtDNA (e.g. (42,57)).

A recent study that claimed bi-parental inheritance of mtDNA in three families suffering mitochondrial disease ((12), see more details below) received crucial comments by multiple groups. Among those, Lutz-Bonengel and Parson criticized that the authors did not exclude NUMTs as source for the observed mtDNA mixtures (58). In another response Balciuniene & Balciunas suggested the presence of ‘multi-copy mtDNA concatemers’, which they termed ‘Mega-NUMTs’ (13).

In this study, we provide strong evidence for the presence of Mega-NUMTs in eight healthy, maternally related members of a family pedigree. We came across this biological phenomenon by pure coincidence, as a laboratory trainee, individual IV.3, provided a buccal swab and a blood sample for contamination elimination purposes. Sequencing the mtDNA from blood of this individual resulted in a mixture of two phylogenetically plausible mitogenome sequences corresponding to hgs V and U4c1. The presence of the mixture was also visible in the buccal sample, with the U mitotype being present at a smaller fraction than in blood (Supplementary Figure S3). Cloning analyses confirmed the mixture for both tissues (Figure 2). The number of mitochondria is known to vary between different tissues, whereas each cell contains only one nucleus. This explains the observed differences of the relative contribution of the U mitotype (nDNA) to the V mitotype (mtDNA).

The following considerations suggest the presence of a full mitogenome NUMT as opposed to partial mitogenome sequences. The PCR amplification of the entire human mitogenome by generating two ∼8.5 kb fragments overlapping at both ends is straightforward when starting from a circular template (see Supplementary Figure S1 in (59)). However, with a linear target such as a NUMT sequence, the situation is more complicated. A single, non-repetitive, linearized complete mitogenome would at best allow the amplification of one of the two long-range PCR products generated in this study but not of both. In theory, several other mtDNA derived matrices may be suitable for the long-range PCR results obtained here, e.g. two separate targets containing at least the amplified sequences (positions 2480–10858, 8,378 bp; 10653–16569|1–2688, 8,605 bp) and—based on our results—featuring the same sequence in the two overlapping ∼200 bp regions would be one of these possibilities. Another theoretical possibility would be a linear sequence containing at least the positions 2480–16569|1–2688 (16,777 bp) or the positions 16053–16569|1–10858 (16,774 bp), which would also meet the template requirements of our long-range PCR results. However, instead of postulating two independent templates or a partially elongated mitogenome sequence, the presence of a tandemly repeated complete human mtDNA sequence seems more plausible, as it represents a logic component for a large mtDNA concatemer that has been described earlier (60).

The U mitotype was only observed (in mixture with the V mitotype) in tissues that contained nDNA, i.e. in whole blood, buccal cells, intestinal tissue and hair roots. Tissues that naturally lack intact nDNA, such as single thrombocytes, exclusively yielded the V mitotype. Hair shafts that are known to contain only minute amounts of high molecular weight nDNA (61), also yielded only the V mitotype in 55 of 56 analyzed hair shafts. Only one of the seven hair shaft samples from individual IV.5 showed the U mitotype in addition to the V mitotype. This could be due to some residual amount of nDNA in that particular extract. The hair roots of all affected individuals also contained the U mitotype in mixture with the V mitotype. In some of the hair root samples only the V mitotype was detected, which would be expected by loss or degradation of nDNA as is routinely observed with hair samples in forensic analyses (62).

These experiments were conducted in earlier stages of the study and suggested that the hg U mitotype did not originate from the cells’ mitochondria. In order to test this hypothesis in a more substantiate way, we sequenced ρ^0^ cells, in which mtDNA was depleted. These experiments resulted only in the U mitotype without any detectable traces of the V sequence, even when using sensitive short amplicon MPS. This result provides very strong evidence that the source of the U mitotype was associated with nDNA, while the V mitotype was located within the mitochondria and constitutes the mtDNA in the investigated matriline. Finally, the U mitotype was located to chromosome 14 (14q13) using FISH. Quantitative analyses resulted in 45 to 56 mitogenome copies, which suggests that the nuclear insert is between 700 kbp and 900 kbp in size. The exact mitogenome copy number and its nucleotide sequence might be determined by long-range sequencing in the future.

Mitochondrial DNA sequencing showed the U/V mixture in eight of the eleven maternally related individuals in this pedigree. The maternal great-grandmother (I.2) to our reference individual IV.3, as well as her aunt (III.6) and her cousin (IV.7) harbored only the V mitotype. We therefore conclude that III.6 has inherited (and transmitted) the homologue chromosome 14 lacking the Mega-NUMT insertion. Also, the children of individuals IV.1 and IV.2 (V.1–3), that exclusively showed the hg H24 mitotype from their mother (IV.2) have inherited the unaffected chromosome 14. We were able to obtain a blood sample from child V.2 that confirmed this reasoning by yielding only the H24 mitotype without any traces of U sequences.

From the great-grandmother (I.2) and the great-grandfather (I.1) we obtained bone samples during the re-establishment of the grave site. The bone sample of I.2 yielded only the V mitotype with both direct sequencing as well as sequencing of 249 clones. Also, the bone samples of the great-grandfather (I.1) did not yield any U component. However, one of these two samples (I.1 or I.2) would be expected to contain the U mitotype (either as NUMT or as mtDNA prior to nuclear integration) to explain the presence of the NUMT in individual II.2 and the rest of the pedigree. We exclude false paternity as a possible reason for this finding (see above), but cannot exclude that the generally low amount of nDNA and particularly the degraded nature of nDNA in aged bones from graves may have concealed the U mitotype in generation I. We observed signs of DNA degradation in the STR electropherograms of I.1 and I.2 (data not shown), but can only speculate as to how much the NUMT portion may have been affected by it. A possibility for future investigation of this question would be additional experiments targeting small amplicon (42) or primer capture extension assays (63).

A recent study reported phylogenetically plausible mtDNA mixtures in the blood of three unrelated patients with mitochondrial disorders and in some of the closely related family members (12). These results were explained with biparental inheritance of mtDNA. However, these conclusions were challenged (58,59,64,65) because the authors failed to exclude a significant NUMT contribution to the observed mixture by experimental work such as described herein. There are striking similarities between the study of Luo *et al.* (12) and our study: first, all observed mixtures consisted of full mitogenome sequences that represented modern mtDNA haplogroups. Fixed NUMTs are generally known to consist of ‘archaic’ sequence motifs, by which they can be easily discerned from modern mtDNA (56). Second, the Mega-NUMTs were present at high copy number, which enabled their detection through direct sequencing approaches in diverse tissues. Our results were paralleled in a recent study that identified Mega-NUMT*s* in seven of more than 11,000 investigated trios (31 affected individuals) (60). The authors located the Mega-NUMT*s* to chromosomes 3, 7, 12, 13 and 17. There, only the fathers were found to carry the Mega-NUMT, whereas the results of our study and the study by (12) suggest that also the mothers passed the nuclear insert to the next generation, what would be expected if the NUMT segregated in an autosomal dominant manner regardless of its parental origin ((13); Figure 1 and Figures 1–3 in (12). Therefore, the observed restriction to fathers passing the Mega-NUMT in (60) may relate to their strategy for NUMT detection (see Figure 3 in (60)). In summary, these studies provide evidence that full mitogenome Mega-NUMTs do exist and, depending on the applied allele frequency detection, may be more common than currently thought. Our study for the first time proves the existence of Mega-NUMT*s* by physically separating mtDNA from nDNA through ρ^0^ cells, by locating the nuclear component via FISH and by sequencing tissues that naturally lack intact nuclear DNA (hair shafts and thrombocytes).

In routine practice, e.g. medical counselling after mtDNA screening analysis, co-amplification and sequencing of both targets, i.e. the Mega-NUMT and the genuine mtDNA cannot be avoided in tissues like blood or buccal cells and thus may be mistakenly considered as heteroplasmic events. The generation of ρ° cells or the selective enrichment of mtDNA during DNA extraction, e.g. by CsCl-ethidium bromide density gradient ultracentrifugation, are impractical for routine work. However, we suggest the sequence analysis of hair shafts from the affected individual as an elegant alternative. While hair shafts usually contain sufficient amounts of mtDNA for PCR-based analyses, they typically contain only minute amounts of highly degraded nuclear DNA (61). Consequently, PCR assays aiming at mtDNA target sequences will almost exclusively yield amplicons deriving from the actual mtDNA copies being present in a hair shaft, as demonstrated in our study. Alternatively, the analysis of thrombocytes can be performed that are void of nuclei and thus should only result in genuine mtDNA sequences. Based on our findings, reports on paternal inheritance of mtDNA should now require explicit exclusion of NUMTs as the reason for the observed mixed sequence patterns. We suggest mtDNA sequencing of DNA extracted from hair shafts or thrombocytes and—if feasible—from ρ° cells as a requirement for future studies that claim paternal leakage of mtDNA in humans.

## DATA AVAILABILITY

Mixed control region mitotype sequences were deposited on GenBank under accession numbers MT780570–9. Further sequence data are available upon request.

## Supplementary Material

gkaa1271_Supplemental_File
