# Supplementary material for: Evidence for multi-copy Mega-NUMTs in the human genome
Source: Nucleic Acids Res. 2021 Jan 15;49(3):1517–31. doi: 10.1093/nar/gkaa1271 (PMC7897518; doi:10.1093/nar/gkaa1271)
Supplement: gkaa1271_Supplemental_File [file gkaa1271_supplemental_file.pdf]

Supplementary Information

to: Evidence for multi-copy Mega-NUMTs in the Human Genome

1. Figure S1	2
2. Figure S2	3
2. Figure S3	4
3. Table S1	5
4. Table S2	7
5. Table S3	8
6. Table S4	9

Figure S1. Timeline for the collection of the various tissues examined (buccal cells, blood, intestinal tissue and bones). Asterisks indicate samples that have been examined independently in laboratory 1 (Institute of Forensic Medicine, Freiburg) and 2 (Institute of Legal Medicine, Innsbruck), with the analyses starting from DNA extraction in each case. The blood collection on 07.06.2011 was performed by an independent company using its own materials.

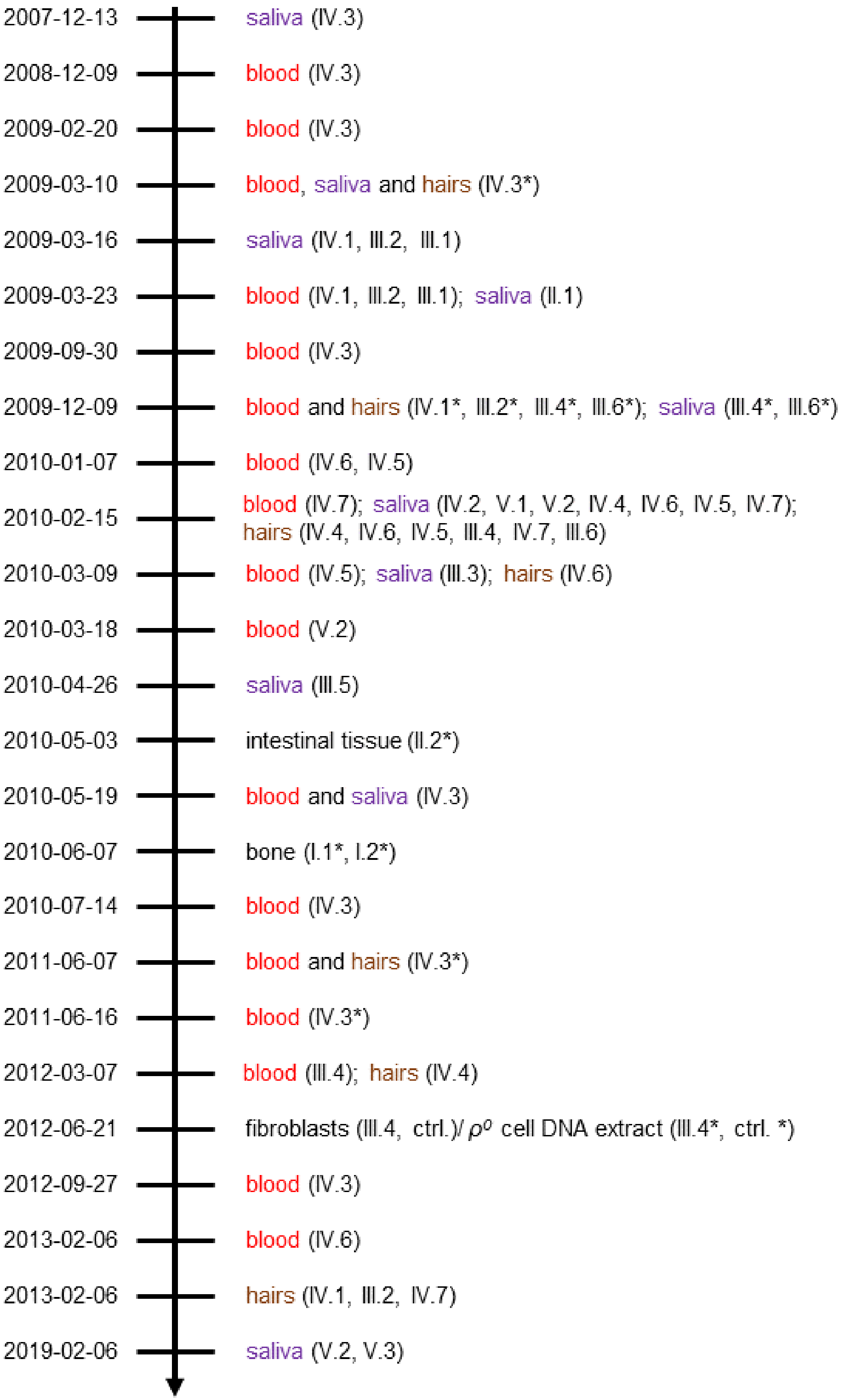

Figure S2. Display of an exemplar autosomal Short Tandem Repeat (STR) profile showing a single source STR genotype for individual IV.3. 15 STR systems (plus amelogenin for sex determination) were analyzed revealing a maximum of two alleles per system (single source genetic profile) excluding external DNA contamination and chimerism as sources for the observed mtDNA mixture.

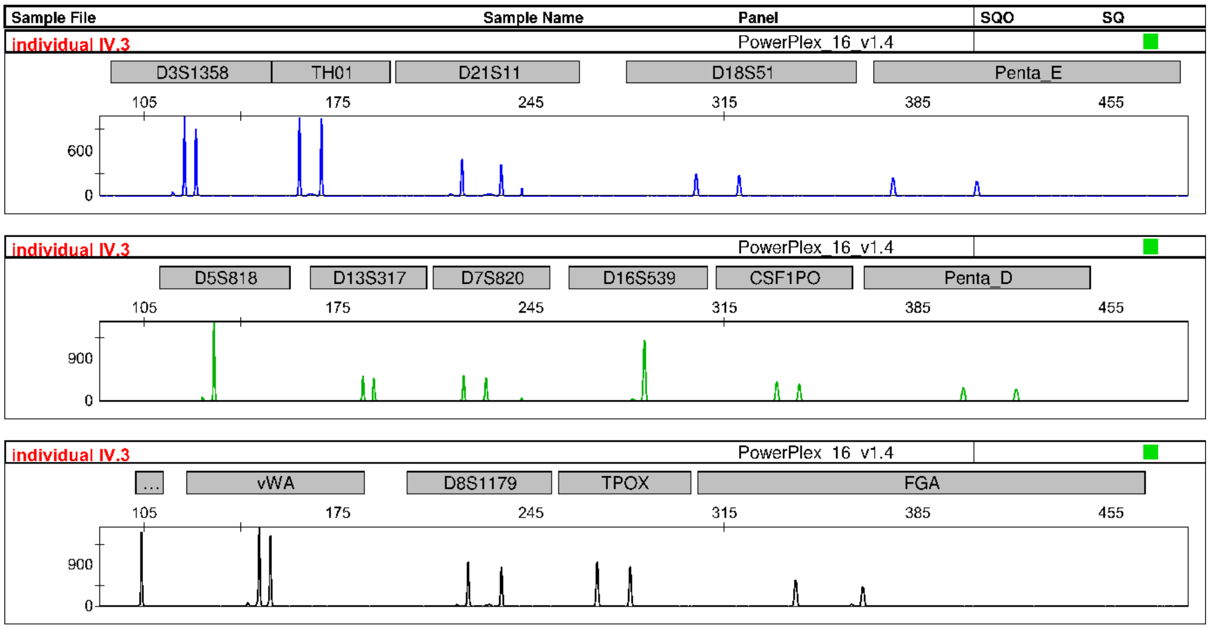

Figure S3. Representative Sanger raw sequence data of the mtDNA control region (rCRS positions 187–202) from seven maternally related family members that display the mixed haplotype and from two maternally related family members (IV.7, III.6) with a neat haplotype in buccal cells (left) and in blood (right). Ind: individual, ntp: nucleotide position within the mitochondrial genome.

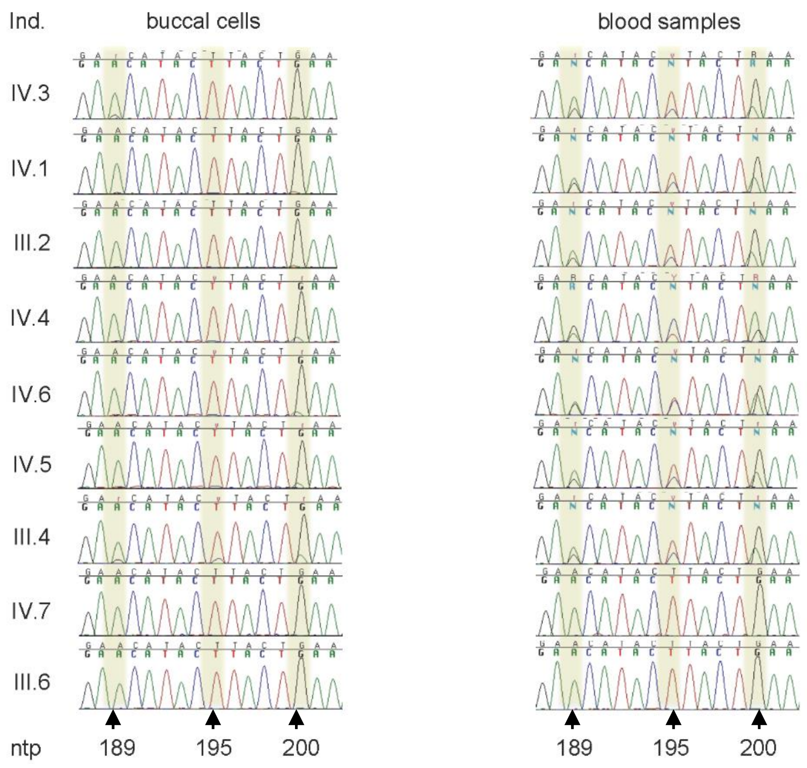

Table S1. List of experiments sorted by individual and tissue/cell type.
lab1: Institute of Forensic Medicine, Freiburg; lab2: Institute of Legal Medicine, Innsbruck; lab3: Institute of Human Genetics, Jena; CR: mitochondrial control region; ddPCR: droplet digital PCR, FISH: fluorescence in situ hybridization; MPS: massively parallel sequencing; qPCR: real-time quantitative PCR; STR: short tandem repeat; STS: Sanger-type sequencing.

**Family member**	**Sample**	**STS of mtDNA CR**	**STR genotyping**	**Mitogenome sequencing (MPS)**	**Cloning**	**FISH**	**ddPCR**	**qPCR**	**Short amplicon MPS on hair shafts**	**STS of full mtDNA long-range PCR amplification**
**involved laboratory**		lab1, lab2	lab1, lab2	lab2	lab1	lab3	lab2	lab2	lab2	lab2
**I.1**	bone	X	X		X					
**I.2**	bone	X	X		X					
**II.1**	buccal cells	X	X							
**II.2**	paraffin embedded intestinal tissue	X	X		X					
	blood (EDTA)	X			X					
	buccal cells	X	X		X					
	hair	X							X	
**III.2**	PBMC	X								
	blood (EDTA)	X			X		X			
	buccal cells	X	X		X					
	hair	X							X	
	PBMC	X								
**III.4**	ρ^0 cells	X					X	X		X
	blood (EDTA)	X			X					
	buccal cells	X	X		X					
	PBMC	X								
**III.6**	hair	X							X	
**III.1**	blood (EDTA)	X								
	buccal cells	X	X							
**III.3**	buccal cells	X	X							

Family member	Sample	STS of mtDNA CR	STR genotyping	Mitogenome sequencing (MPS)	Cloning	FISH	ddPCR	qPCR	Short amplicon MPS on hair shafts	STS of full mtDNA long-range PCR amplification
**III.5**	buccal cells	X								
	blood (EDTA)	X	X	X	X					X
	buccal cells	X	X		X					
	hair	X							X	
	PBMC	X								
	thrombozytes	X								
**IV.3**	blood (Heparin)					X				
	blood (EDTA)	X			X					
	buccal cells	X	X		X					
	hair	X							X	
**IV.1**	PBMC	X								
	blood on cellulose	X	X		X					
**IV.4**	buccal cells	X	X		X					
	hair	X							X	
	blood on cellulose	X			X					
**IV.6**	buccal cells	X	X		X					
	hair	X							X	
	blood on cellulose	X			X					
**IV.5**	buccal cells	X	X		X					
	hair	X							X	
	blood on cellulose	X			X					
**IV.7**	buccal cells	X	X		X					
	hair	X							X	
**VI.2**	buccal cells	X								

Family member	Sample	STS of mtDNA CR	STR genotyping	Mitogenome sequencing (MPS)	Cloning	FISH	ddPCR	qPCR	Short amplicon MPS on hair shafts	STS of full mtDNA long-range PCR amplification
**V.1**	buccal cells	X								
**V.2**	blood on cellulose	X	X							
	buccal cells	X								
**V.3**	buccal cells	X								

Table S2. Sequences of mtDNA amplification and sequencing primers.

primerID	oligonucleotide sequence	rCRS position
F15	CACCCTATTAACCACTCACG	15 → 34
F29	CTCACGGGAGCTCTCCATGC	29 → 48
R159	AAATAATAGGATGAGGCAGGAATC	136 ← 159
R381	GCTGGTGTTAGGGTTCTTTG	362 ← 381
R599	TTGAGGAGGTAAGCTACATAA	579 ← 599
R639	GGGTGATGTGAGCCCGTCTA	620 ← 639
F15851	ATCTCCCTAATTGAAAACAAAATACTCAAA	15851 → 15880
F15900	TAAACTAATACACCAGTCTTGTAAACC	15900 → 15926
F16268	CACTAGGATACCAACAAACC	16268 → 16287

rCRS: revised Cambridge Reference Sequence [30]

Table S3. MtDNA control region sequences of family members outside the maternal pedigree.

Individuals	Haplotype (CR)	Haplogroup
V.1, V.2, V.3, IV.2	16293G 16519C 263G 315.1C	H24
III.1	16069T 16126C 16145A 16231C 16261T 73G 150T 152C 195C 215G 263G 295T 310.1T 315.1C 319C 489C 513A	J2a1a1a
III.3	263G 315.1C	H/R0
III.5	16519C 263G 309.1C 573.1C 573.2C 573.3C	H/R0
II.1	16136C 16242T 16356C 16519C 73G 195C 263G 309.1C 315.1C 499A 524.1A 524.2C	U4b2
I.1	315.1C	H/R0

CR: mitochondrial control region (revised Cambridge Reference Sequence [30], positions 16,024–576)

Table S4. Summary of mitogenome sequencing results (MPS) from hair shaft samples of nine maternally related individuals, seven of which harbored the Mega-NUMT and two (III.6, IV.7) did not. The table shows that in hair shafts only the V haplotype was observed and no U contribution that was found in other tissues of the seven affected individuals, because hair shafts do not contain enough nuclear DNA to detect the Mega-NUMT.

	**# 1-16569**								
	**mean coverage per sample**								
	**8367**	**10858**	**10754**	**9815**	**13544**	**11298**	**10952**	**10950**	**8218**
**V**	**IV.3**	**IV.1**	**III.2**	**III.4**	**III.6**	**IV.4**	**IV.5**	**IV.6**	**IV.7**
**variant**	**variant calling [%]**								
72C	100	100	100	100	100	56 (43 T)	100	100	100
200G	99	99	98	99	99	99	99	99	99
263G	100	100	100	100	99	100	99	100	100
309.1C	NA	NA	NA	NA	NA	NA	NA	NA	NA
315.1C	NA	NA	NA	NA	NA	NA	NA	NA	NA
750G	100	100	100	100	100	100	100	100	100
1438G	100	100	100	100	100	100	100	100	100
2706G	100	100	100	100	100	100	100	100	100
4580A	100	100	100	100	99	100	99	100	100
4769G	100	100	100	100	100	100	100	100	100
7028T	100	100	100	100	100	100	100	100	100
8134C	100	100	100	99	100	100	99	100	100
8860G	100	100	100	100	100	100	99	100	100
15326G	100	100	100	100	100	100	100	100	100
15904T	100	100	100	100	100	100	100	100	100
16298C	100	100	100	99	100	100	100	100	99
